# Efficacy and safety of goniotomy and gonioscopy-assisted transluminal trabeculotomy for exfoliation glaucoma: a systematic review and single-arm meta-analysis

**DOI:** 10.3389/fmed.2026.1718315

**Published:** 2026-03-11

**Authors:** Weijia Li, Yulei Geng, Kuitang Shi, Guangxian Tang, Xiaowei Yan, Yawen Li, Tianyu Zhang, Jiaming Lu, Shuai Wang, Hengli Zhang

**Affiliations:** Shijiazhuang People's Hospital, Shijiazhuang, China

**Keywords:** exfoliation glaucoma, GATT, goniotomy, meta-analysis, minimally invasive glaucoma surgery

## Abstract

**Objective:**

To assess the efficacy and safety of goniotomy (GT) and gonioscopy-assisted transluminal trabeculotomy (GATT) with or without phacoemulsification (PEI) for exfoliation glaucoma (XFG).

**Methods:**

Searches were conducted in PubMed, Scopus, Embase, Ovid, and the Web of Science. Two independent reviewers performed study selection, data extraction, and quality assessment. The primary outcomes were the reduction in intraocular pressure (IOP) and the number of antiglaucoma medications (AGMs) at 1, 6, and 12 months postoperatively. Safety was assessed by the incidence of complications.

**Results:**

Fourteen studies involving 624 eyes were included. The GATT ± PEI group showed significant IOP reductions of 1.96 mmHg (*p* < 0.01), 2.17 mmHg (*p* < 0.001), and 2.07 mmHg (*p* < 0.001) at 1, 6, and 12 months, respectively, with corresponding AGM reductions of 3.28 (*p* < 0.001), 2.87 (*p* = 0.003), and 2.54 (*p* = 0.011). The GT + PEI group demonstrated IOP reductions of 1.95 (*p* < 0.001), 2.00 (*p* = 0.040), and 2.13 mmHg (*p* = 0.013), with AGM reductions of 1.07, 0.96, and 0.96 (all *p* < 0.001). For standalone procedures, GT significantly reduced AGMs at all timepoints (all *p* < 0.001) and IOP at 1 and 6 months (both *p* = 0.002), while standalone GATT significantly reduced IOP only at 1 month (2.39 mmHg, *p* < 0.001) and AGMs at 6 and 12 months (both *p* < 0.001). The most common complications were anterior chamber hemorrhage (47.6%) and IOP spike (17.0%).

**Conclusion:**

This meta-analysis demonstrates that GT and GATT, particularly when combined with phacoemulsification, are safe and effective in reducing IOP and medication burden in patients with XFG. The evidence for sustained IOP lowering with standalone GATT remains limited, suggesting that combined surgery may offer more reliable long-term pressure control, especially for patients with concurrent cataract.

**Systematic review registration:**

https://www.crd.york.ac.uk/prospero/display_record.php?ID=CRD420251072295, identifier CRD420251072295.

## Introduction

Glaucoma is the leading cause of irreversible blindness worldwide, and the number of glaucoma patients is expected to exceed 100 million by 2040 (1). Exfoliative glaucoma (XFG), a significant subtype, has a poorer prognosis than primary open-angle glaucoma (POAG), characterized by rapid progression, marked intraocular pressure (IOP) fluctuations, and more severe optic nerve damage at presentation (2, 3).

Lowering IOP remains the only effective strategy for slowing XFG progression. Medication often fails to achieve target levels, whereas traditional drainage procedures (e.g., trabeculectomy) carry high risks of complications (e.g., shallow anterior chamber, choroidal detachment) and long-term drainage-related complications (4).

In recent years, minimally invasive glaucoma surgery (MIGS) has emerged as a novel surgical approach bridging the gap between medical therapy and drainage procedures (5). MIGS offers advantages, including high safety, rapid recovery, a short operative time, and low complication rates, providing a new treatment option for glaucoma (6, 7). MIGS is typically combined with phacoemulsification. For patients with glaucoma and cataracts, combined surgery yields better visual acuity improvement and intraocular pressure reduction than cataract extraction alone.

This meta-analysis focuses on two specific MIGS procedures: goniotomy (GT) and gonioscopy-assisted transluminal trabeculotomy (GATT). GT involves a direct incision of the trabecular meshwork under gonioscopic guidance. GATT utilizes an illuminated microcatheter or a suture to circumferentially cannulate and tear through Schlemm’s canal, potentially offering a more extensive ablation of the outflow resistance (8–10).

Although GT and GATT’s efficacy in POAG is established, their effectiveness in XFG remains unclear (11, 12). Therefore, this study aimed to evaluate GT and GATT’s efficacy and safety for treating XFG through a systematic review and meta-analysis while comparing the therapeutic differences among various surgical approaches.

## Methods

This meta-analysis adhered to the 2020 Preferred Reporting Items for Systematic Review and Meta-Analysis (PRISMA) guidelines and was registered in the PROSPERO database (registration number: CRD420251072295) on June 1, 2025. Two mutually blinded authors (W.L. and K.S.) independently conducted the title and abstract screenings and article assessment. The senior author (H.Z.) resolved disagreements.

### Search strategy

The literature search encompassed five electronic databases: (1) PubMed; (2) Ovid; (3) Web of Science; (4) Scopus; and (5) Embase. The most recent search was conducted on June 13, 2025. The search strategy combined Medical Subject Headings (MeSH) terms and free-text terms related to the target population (e.g., “exfoliation glaucoma,” “pseudoexfoliation glaucoma”) and interventions [e.g., “goniotomy,” “trabeculotomy,” “Gonioscopy-assisted transluminal trabeculotomy (GATT)”]. Additionally, references from the retrieved articles were screened to identify further eligible studies.

### Inclusion and exclusion criteria

The inclusion criteria were as follows: (1) the study subjects were diagnosed with exfoliation glaucoma; (2) the interventions included GT or GATT; and (3) the research outcomes included IOP, the number of AGMs, and complications, which can be directly obtained from the literature.

The exclusion criteria were as follows: (1) reviews, editorials, case reports, meta-analyses, letters, animal studies, guidelines, conference abstracts, and opinion articles lacking original data; (2) mixed glaucoma studies not reporting separate data for exfoliation glaucoma (XFG); (3) follow-up periods shorter than 1 year; (4) overlapping study populations; and (5) non-English-language literature.

### Data collection

Two researchers independently extracted data via standardized Excel templates. The extracted data included the first author, publication year, study design, sample size, demographic characteristics (country, age, sex), follow-up duration, surgical instrument, mean ± SD of IOP, and number of AGMs. The primary outcomes were baseline and postoperative (1/6/12 months) IOP and the number of AGMs. All reported complications were documented. It is acknowledged that variations in measurement protocols (e.g., time of day for IOP assessment) exist across studies, which constitutes a source of clinical heterogeneity.

### Quality assessment

The Newcastle–Ottawa Scale (NOS) was used to evaluate the quality of the included cohort studies, specifically for assessing participant selection, group comparability, and outcome measurement. Studies scoring between 7 and 9 were categorized as “High,” those scoring between 4 and 6 were deemed “fair,” and studies with scores below 3 were labelled “poor” (11).

### Statistical analysis

STATA 18.0 was used for the meta-analysis. The mean differences and 95% confidence intervals (CIs) for preoperative versus postoperative IOP and the number of AGMs were calculated. Complications not explicitly reported were considered absent. Subgroup analyses were performed to compare surgical approaches and devices. Heterogeneity was assessed using the I^2^ statistic and Cochran’s Q test. A random-effects model was used if significant heterogeneity was present (I^2^ > 50% and Q-test *p* < 0.10); otherwise, a fixed-effects model was applied. Sensitivity analyses employed sequential exclusion methods, and publication bias was assessed via Egger’s test.

## Results

### Search characteristics

Initially, 4,837 potentially eligible studies were identified, and articles not meeting the specified eligibility criteria were excluded. Following the screening process, 14 articles involving a combined total of 624 eyes were selected for the final analysis.

**Figure 1:**
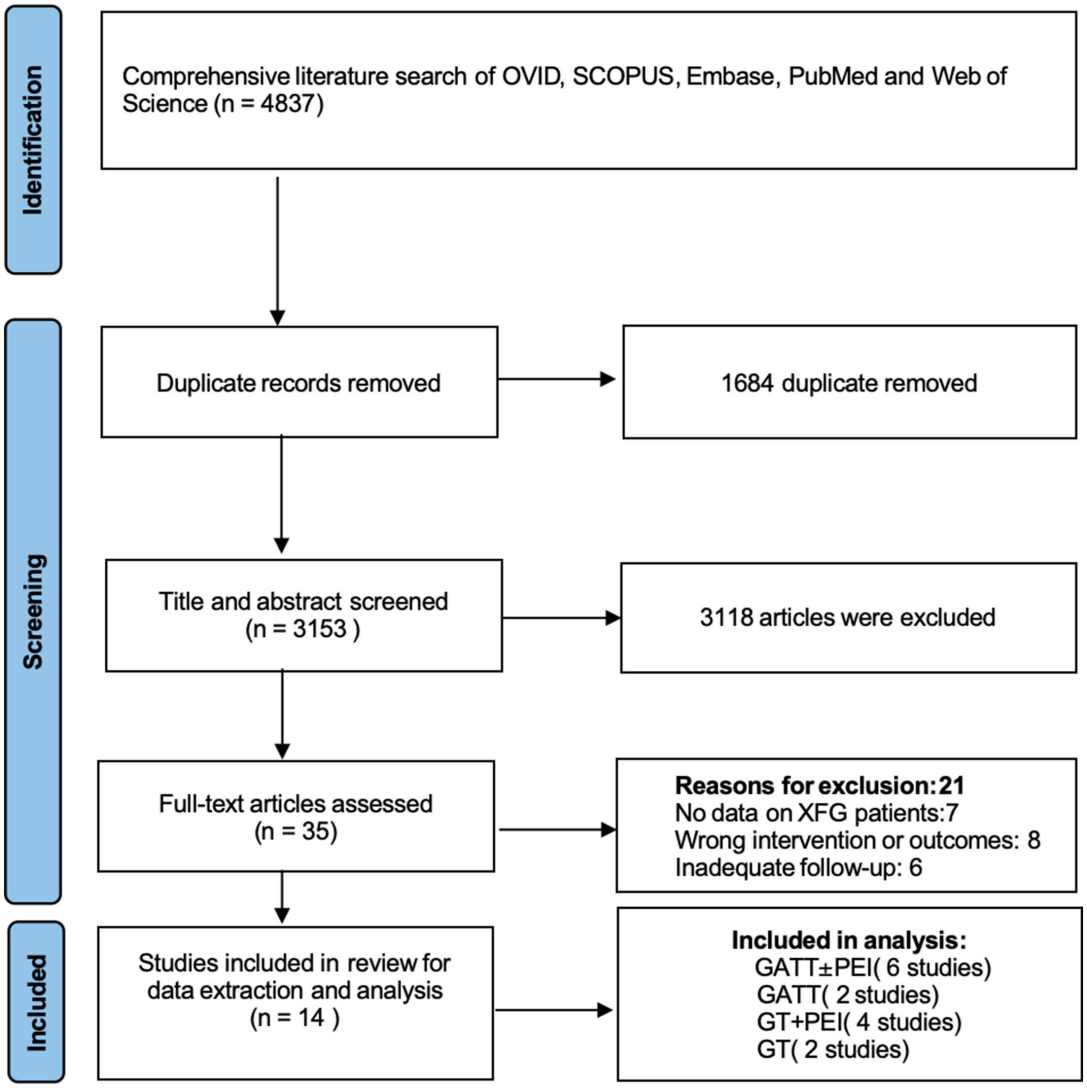
Flow chart of study selection. GT, goniotomy; GATT, gonioscopy-assisted transluminal trabeculotomy; XFG, exfoliation glaucoma; PEI, phacoemulsification with intraocular lens implantation.

Among these, 6 studies reported outcomes for GATT ± PEI, 2 for standalone GATT, 2 for standalone GT, and 4 for GT + PEI (Figure 1). The baseline characteristics of the included studies are shown in Table 1.

**Table 1:** Characteristics of the enrolled patients.

Author, Year	Country	No. of eyes	Age (years)	Preoperation medications	Baseline IOP (mmHg)	Quality evaluation
GATT ± PEI						
Hepşen et al. (17), 2016	Turkey	20	63.15 ± 6.78	3.15 ± 0.81	26.55 ± 8.91	Good
Bozkurt et al. (13), 2021	Turkey	66	68.91 ± 12.30	2.94 ± 1.21	25.35 + 8.52	Good
Cubuk et al. (14), 2020	Turkey	22	68.20 ± 7.00	3.50 ± 0.50	27.50 ± 10.00	Good
Aktas et al. (5), 2022	Turkey	111	72.00 ± 10.10	3.10 ± 0.80	26.10 ± 7.40	Good
Sharkawi et al. (20, 28), 2021	Turkey	103	75.50 ± 8.90	2.90 ± 1.10	27.10 ± 11.7	Good
Gunay et al. (12, 22), 2024	Turkey	31	70.70 ± 6.90	3.60 ± 0.60	29.43 ± 7.18	Good
GATT						
Cubuk et al. (19), 2021	Turkey	14	65.30 ± 4.90	3.80 ± 0.40	24.60 ± 4.50	Good
Gunay (22)	Turkey	31	68.60 ± 10.30	3.80 ± 0.50	28.00 ± 7.30	Good
GT						
Pahlitzsch et al. (18), 2017	Canada	22	–	2.55 ± 0.96	20.86 ± 6.05	Good
Tanihara et al. (23), 1993	Japan	17	59.50 ± 11.80	–	30.80 ± 8.40	Good
GT+ PEI						
Fukuchi et al. (24), 2011	Japan	33	76.20 ± 4.23	3.21 ± 1.58	22.40 ± 5.13	Good
Koylu et al. (15), 2025	Turkey	33	69.40 ± 5.90	3.70 ± 0.50	27.10 ± 7.90	Good
Honjo et al. (21), 1998	Japan	49	75.70 ± 6.00	1.60 ± 1.00	24.50 ± 4.80	Moderate
Iwasaki et al. (16) 2022	Japan	38	79.00 ± 6.20	2.70 ± 1.50	22.40 ± 7.40	Good

### Reduction of IOP and AGM

In the GATT ± PEI group, IOP decreased by 1.96 (*p* < 0.01), 2.17 (*p* < 0.001), and 2.07 (*p* < 0.001) at 1, 6, and 12 months post-operatively, respectively. The number of AGMs decreased by 3.28 (*p* < 0.001), 2.87 (*p* = 0.003), and 2.54 (*p* = 0.011) at 1, 6, and 12 months post-operatively, respectively.

In the GATT group, IOP decreased by 2.39 (*p* < 0.001), 2.52 (*p* = 0.711), and 2.65 (*p* = 0.821) at 1, 6, and 12 months post-operatively, respectively. The number of AGMs decreased by 3.11 (*p* < 0.001) and 3.94 (p < 0.001) at 6 and 12 months post-operatively, respectively.

In the GT group, the IOP decreased by 1.27 (*p* = 0.002), 1.57 (*p* = 0.002), and 1.23 (*p* = 0.492), respectively. AGM counts reduced by 1.00 (*p* < 0.001), 1.04 (*p* < 0.001), and 0.54 (*p* < 0.001) at 1, 6, and 12 months post-operatively, respectively.

**Figure 2:**
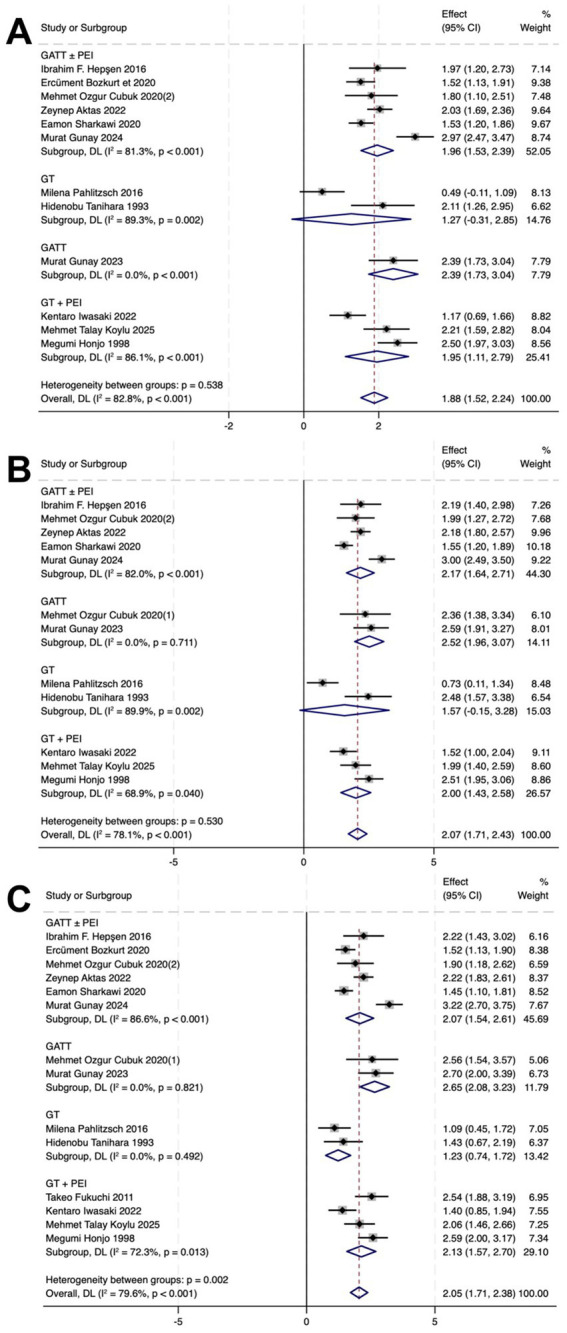
Forest plots showing postoperative and preoperative IOP at 1 **(A)**, 6 **(B)**, and 12 **(C)** months. CI, confidence interval.

**Figure 3:**
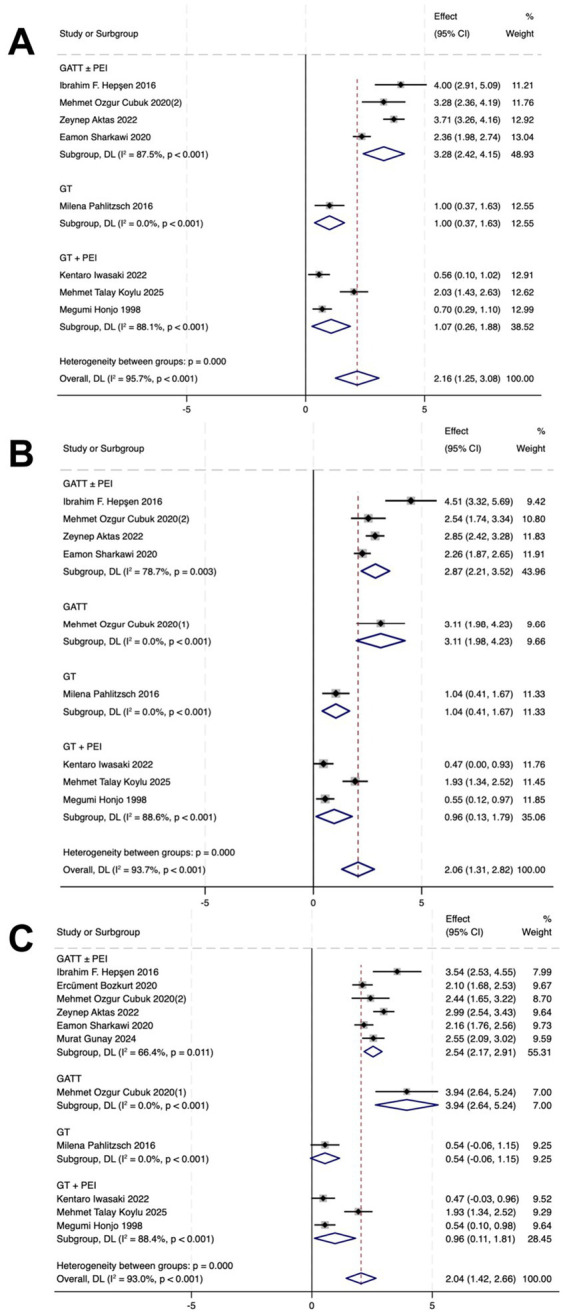
Forest plots showing postoperative and preoperative the numbers of AGMs at 1 **(A)**, 6 **(B)**, and 12 **(C)** months. CI = confidence interval.

In the GT + PEI group, the IOP decreased by 1.95 (*p* < 0.001), 2.00 (*p* = 0.040), and 2.13 (*p* = 0.013), respectively. AGM counts reduced by 1.07 (*p* < 0.001), 0.96 (*p* < 0.001), and 0.96 (*p* < 0.001) at 1, 6, and 12 months post-operatively, respectively (Figures 2, 3).

### Influence of surgical device in the GATT ± PEI cohort

In the suture-based GATT subgroup, IOP decreased by 2.31 mmHg (*p* < 0.001) at 12 months postoperatively. AGM counts reduced by 2.76 (*p* = 0.008) at 12 months postoperatively.

**Figure 4:**
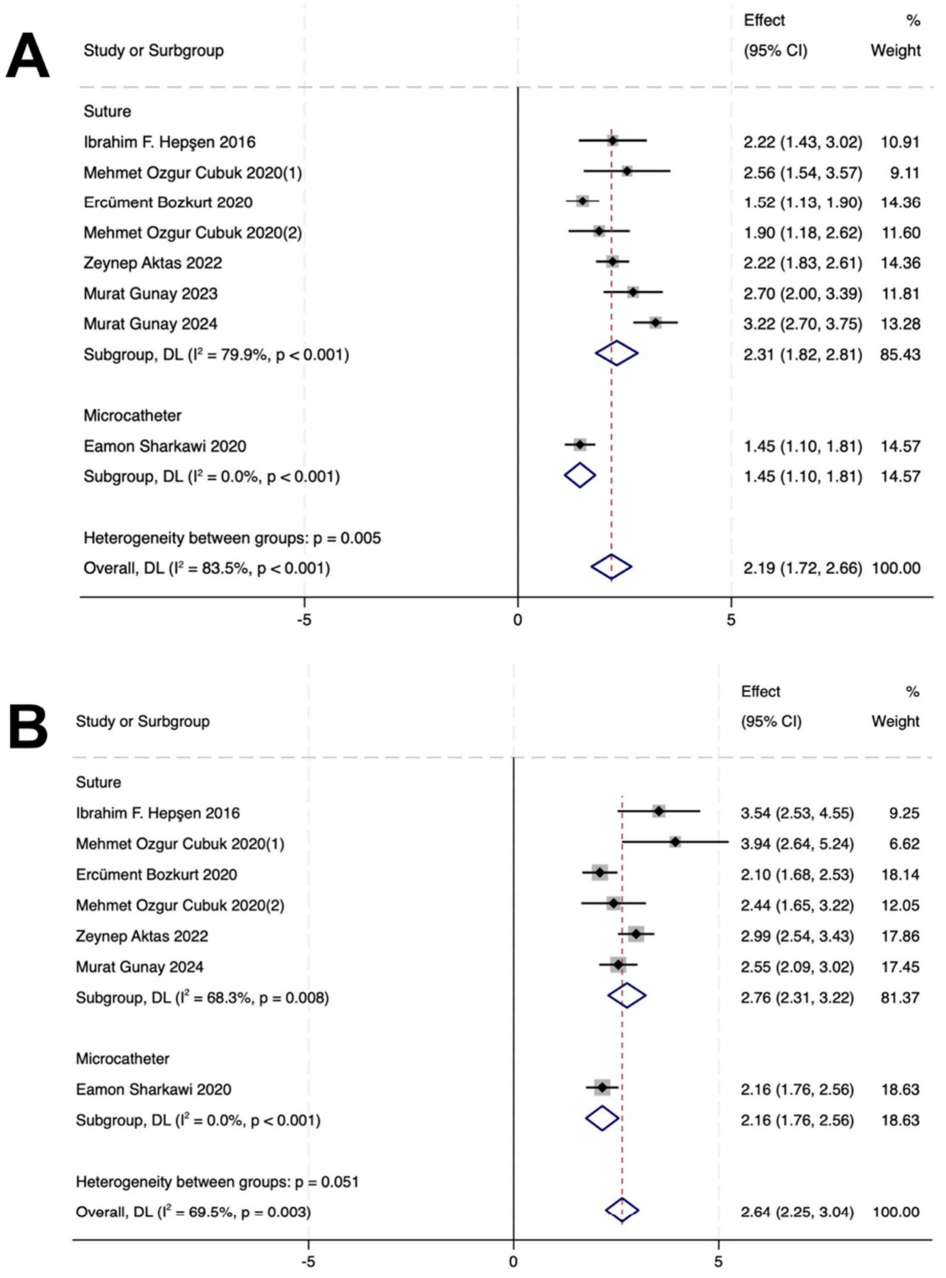
Forest plots of subgroup analyses comparing surgical devices (suture vs. microcatheter) for GATT procedures within the GATT ± PEI cohort. **(A)** Mean difference in intraocular pressure (IOP) reduction from baseline at 12 months postoperatively. **(B)** Mean difference in the number of antiglaucoma medications (AGMs) reduced from baseline at 12 months postoperatively.

In the microcatheter-based GATT subgroup, IOP decreased by 1.45 mmHg (*p* < 0.001) at 12 months postoperatively. AGM counts reduced by 2.16 (*p* < 0.001) at 12 months postoperatively (Figure 4).

### Safety

Table 2 shows the most common complications. Among the 624 eyes, 297 (47.6%) developed anterior chamber hemorrhage, and 106 (17.0%) experienced an IOP spike. The GATT± PEI group had a higher incidence of anterior chamber hemorrhage (35.8% of total eyes) and a higher incidence of IOP spike (9.9% of total eyes).

**Table 2:** The main complication.

Author, Year	Hyphema	IOP spike
Total	**297 (47.6)**	**106 (17.0)**
GATT ± PEI	**224 (35.8)**	**62 (9.9)**
Hepşen et al. (17), 2016	20	1
Aktas et al. (5), 2022	36	15
Gunay et al. (12, 22), 2024	65	21
Sharkawi et al. (20, 28), 2021	103	25
GATT	**22 (3.5)**	**6 (0.9)**
Gunay (22)	22	6
GT	**9 (1.4)**	**0 (0)**
Tanihara et al. (23), 1993	9	0
GT + PEI	**42(6.7)**	**38 (6.1)**
Fukuchi et al. (24), 2011	30	10
Koylu et al. (15), 2025	3	8
Honjo et al. (21), 1998	3	11
Iwasaki et al. (16) 2022	6	9

The bold values in this table represent the total number (n) and percentage (%) of complications for each surgical subgroup (Total, GATT ± PEI, GATT, GT, GT + PEI).

### Publication bias and sensitivity analysis

**Figure 5:**
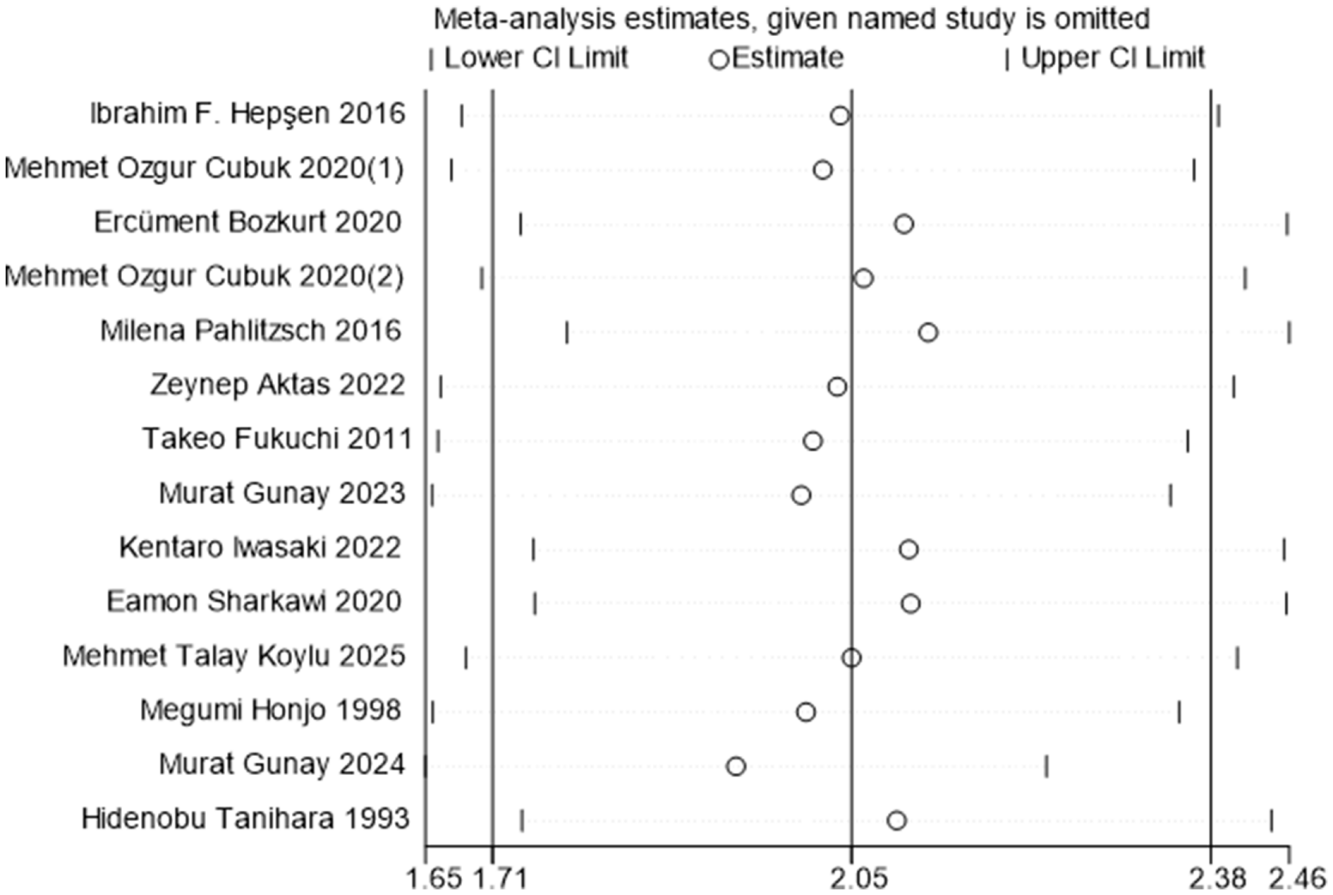
The sensitivity analysis result.

Given the significant heterogeneity in the IOP and AGM analyses, postoperative 12-month IOP outcomes were used as a sensitivity analysis indicator. According to Egger’s test, no publication bias existed in the studies that were reported (*p* = 0.310). Sensitivity analysis showed that deleting any one study would not change the significance of the results, which indicates the stability of the meta-analysis (Figure 5).

## Discussion

MIGS for treating exfoliation glaucoma has gained increasing attention (5, 12–24). This study comprehensively evaluated the efficacy and safety of goniotomy (GT) and gonioscopy-assisted transluminal trabeculotomy (GATT) in treating exfoliation glaucoma (XFG) through a systematic review and meta-analysis. The pooled analyses demonstrated significant reductions in both IOP and medication use across the studied procedures.

Subgroup analysis compared the efficacy of different surgical methods. The results indicate that procedures combined with phacoemulsification (GATT ± PEI and GT + PEI) demonstrated significant and sustained IOP reduction throughout the 12-month follow-up. While the standalone procedures (GATT and GT) significantly reduced the number of medications, the evidence for long-term IOP reduction was less consistent. Specifically, for standalone GATT, while a significant IOP reduction was observed at 1 month, the reductions at 6 and 12 months did not reach statistical significance in our model (*p* > 0.05). This suggests that the long-term IOP-lowering efficacy of standalone GATT in XFG remains uncertain based on current evidence and requires further investigation. The more consistent results observed in the combined surgery groups suggest that coupling GATT or GT with phacoemulsification may provide more stable IOP control for XFG patients with concurrent cataract.

Although GATT covers a wider range than GT, our subgroup analysis showed that in the combined surgery group, the magnitude of IOP reduction was not significantly different between GATT ± PEI and GT + PEI at 12 months. Previous studies comparing the efficacy of trabeculotomy with or without PEI for POAG across different incision angles reported similar IOP control outcomes for 120°, 240°, and 360° incisions, which is consistent with the present findings (25, 26).

When comparing the two primary devices used for GATT within the GATT ± PEI cohort, suture-based procedures were associated with a statistically greater reduction in IOP at 12 months compared to microcatheter-based procedures (between-subgroup *p* = 0.005). This suggests that the mechanical properties of the suture may facilitate a more complete or consistent trabeculotomy, potentially leading to enhanced aqueous outflow in eyes with XFG. However, this interpretation must be treated with considerable caution. The microcatheter subgroup contained only a single study, and the suture subgroup exhibited considerable heterogeneity, indicating other unmeasured factors (e.g., surgeon experience) are also influential. Therefore, while this finding highlights surgical device as a potentially critical variable for optimization, it remains hypothesis-generating. Future prospective studies or randomized trials designed to directly compare these devices are warranted to confirm this observed difference and guide surgical technique selection.

In the results on complications, the overall incidence of anterior chamber hemorrhage was 47.6%, which is similar with previous studies of MIGS for primary angle-closure glaucoma (PACG) (27). Notably, the GATT± PEI group presented a greater incidence of anterior chamber hemorrhage, possibly due to increased exposure of the collection duct and scleral venous connections during this procedure (28). This group also presented a greater incidence of IOP spike than did the other group. This could be caused by corticosteroid-induced IOP spike, postoperative opening of the trabecular meshwork and Schlemm’s canal walls, and a greater contact area between corticosteroids and target cells, leading to resistance in aqueous outflow in steroid-sensitive patients (29–31).

This study has several limitations. First, the majority of the included studies were observational cohort designs rather than randomized controlled trials (RCTs). This predominance of lower-level evidence limits the strength of our conclusions, as non-random assignment in these studies is prone to selection bias and confounding. Therefore, the findings should be interpreted as representing effectiveness in real-world settings, with the need for confirmation from future high-quality RCTs. Second, Over 60% of the included eyes were from studies conducted in Turkey. Given the known geographic variation in the epidemiology and phenotypic expression of exfoliation syndrome, the findings from this predominantly Turkish cohort may not be fully generalizable to other populations with potentially different risk profiles and disease courses. This limits the external validity of our pooled results. Third, surgical technique heterogeneity (e.g., resection extent, combined cataract surgery) could not be fully explained by subgroup analysis. Fourth, significant variation in follow-up durations across studies limited the assessment of long-term efficacy. Finally, Variations in the timing of IOP measurements (which typically ignored diurnal variation) may introduce measurement bias and affect the precision and comparability of the pooled estimates.

## Conclusion

This systematic review and meta-analysis demonstrated that angle-based MIGS (GT and GATT), particularly when combined with phacoemulsification, can safely and effectively reduce IOP and medication use in XFG. However, the evidence for sustained IOP lowering with standalone GATT is limited, highlighting the need for further research and careful patient selection.

## Data Availability

The datasets used or analysed during the current study are available from the corresponding author on reasonable request.
